# Determinants of adaptation choices to climate change by sheep and goat farmers in Northern Ethiopia: the case of Southern and Central Tigray, Ethiopia

**DOI:** 10.1186/s40064-016-3042-3

**Published:** 2016-10-01

**Authors:** Fikeremaryam Birara Feleke, Melaku Berhe, Getachew Gebru, Dana Hoag

**Affiliations:** 1 Department of Natural Resource Economics and Management, College of Dryland Agriculture and Natural Resource, Mekelle University, P.O. Box 231, Mekelle, Ethiopia; 2 MARIL Research and Development, P.O. Box 90112, Addis Ababa, Ethiopia; 3 Department of Agricultural and Resource Economics, Colorado State University, Fort Collils, CO 80523-1172 USA

**Keywords:** Sheep, Goat, Climate change, Adaptation, Choice, Determinants, Agro-ecological settings

## Abstract

The livestock sector serves as a foremost source of revenue for rural people, particularly in many developing countries. Among the livestock species, sheep and goats are the main source of livelihood for rural people in Ethiopia; they can quickly multiply, resilient and are easily convertible to cash to meet financial needs of the rural producers. The multiple contributions of sheep and goat and other livestock to rural farmers are however being challenged by climate change and variability. Farmers are responding to the impacts of climate change by adopting different mechanisms, where choices are largely dependent on many factors. This study, therefore, aims to analyze the determinants of choices of adaptation practices to climate change that causes scarcity of feed, heat stress, shortage of water and pasture on sheep and goat production. The study used 318 sample households drawn from potential livestock producing districts representing 3 agro-ecological settings. Data was analyzed using simple descriptive statistical tools, a multivariate probit model and Ordinary Least Squares (OLS). Most of the respondents (98.6 %) noted that climate is changing. Respondents’ perception is that climate change is expressed through increased temperature (88 %) and decline in rainfall (73 %) over the last 10 years. The most commonly used adaptation strategy was marketing during forage shock (96.5 %), followed by home feeding (89.6 %). The estimation from the multivariate probit model showed that access to information, farming experience, number of households in one village, distance to main market, income of household, and agro-ecological settings influenced farmers’ adaptation choices to climate change. Furthermore, OLS revealed that the adaptation strategies had positive influence on the household income.

**Electronic supplementary material:**

The online version of this article (doi:10.1186/s40064-016-3042-3) contains supplementary material, which is available to authorized users.

## Background

Climate change is a global phenomenon that results in global warming, droughts, flooding and depletion of natural resources (Adger et al. 2003; Parry et al. 2004; Naqvi and Sejian 2011). A study by Nelson et al. (2009) indicated that climate change is expected to bring about significant yield losses between 3 and 30 % and extinction of land plants and animal species between 15 and 37 % by 2050 unless remedial measures are taken into consideration. Developing countries are highly vulnerable to climate change since their economy predominantly relies on rain-fed agriculture that totally depends on natural factors. Traditional farming systems practiced, which have low technological capacity, cannot help to adapt and mitigate drastic climate change (Tubiello 2012).

Being a developing country, Ethiopia’s agriculture contributes about 42–45 % to its gross domestic product, employs more than 80 % of the population and generates more than 85 % of foreign exchange earnings (Deressa 2007; Gebreegziabher et al. 2011; You and Ringler 2011). By 2020 in Ethiopia, however, yields from agriculture could fall by 50 % because of the adverse effects of climate change like rise in temperature, drought, flood, erratic rainfall and others (FDRE 2011). Climate change has been recognized as having potentially severe impacts on livelihood and development (Mengestu 2011). Tigray is one of the nine Regional States in Ethiopia that is being affected by recurrent drought because of both its arid and semi arid nature (Deressa et al. 2008). Consequently, the impacts of climate change and variability remain a serious challenge.

Despite the occurrences of persistent droughts and agriculture failure emanated from climate change in the Tigray region, livestock provides multiple economic and social benefits. Particularly, sheep and goats are easily convertible to cash to meet households’ financial problems such as school fees and agricultural inputs from the sales of live animals and their byproducts (meat, egg, manure etc.). As a result, sheep and goats are considered as assets (as a form of insurance) that require minimum initial investment with quick returns due to fast multiplication (Ayele et al. 2008; Legesse et al. 2008; Amankwah et al. 2012; Musara et al. 2013; Hailu 2014).

Although the benefits from sheep and goats hold great promise, the current level of its contribution to supporting rural livelihoods is low due to climate change related factors. Thermal, nutritional, and water related stresses, and restlessness are some of the consequences of climate change related factors that affect sheep and goat productivity (AL-Haidary 2004; Sevi et al. 2007; Alam et al. 2011; Kandemir et al. 2013; Sejian 2013). Increased incidence of disease and parasitic infection, decreasing trend of feed and fodder resources, low productive and reproductive performance are some of the consequences mainly related to the negative effects of climate change (Henry et al. 2012; Singh et al. 2012). Among the livestock species, sheep and goats are more vulnerable due to their heavily reliance on climate sensitive resources and immobility during flood (Oseni and Bebe 2010), and may not adapt to extreme climate change phenomena such as shortage of fodder, floods and droughts (Tologbonse et al. 2011; Sahoo et al. 2013; Taruvinga et al. 2013). As sheep and goats are owned by the poor section of the rural community who are living in dire poverty, any intervention that improves the productivity of sheep and goats could have positive contribution in reducing the existing poverty in the area.

Adaptation therefore remains one of the policy options to address climatic challenges prevailed on the livestock sector such as on sheep and goats (Deressa et al. 2008; Di Faclo et al. 2011). This has great relevance for developing countries seeking to maintain food security if it is focused to go hand-in-hand with the long-term policy priority among poor farmers (Di Faclo et al. 2011; Tubiello 2012). Their decision to adapt to climate change depends on socio-economic and environmental factors (Taruvinga et al. 2013). Obviously, farmers with the low capacity to adapt are generally the most vulnerable to the negative impacts of climate variability and change. Within the spectrum of livestock versus adaptation methods to climatic change, many researchers have identified important adaptation strategies (Dick et al. 2008; Henry et al. 2012; Singh et al. 2012). Despite significant progress, many questions regarding the prospects for ruminant animals mainly of sheep and goats have yet to be recognized (Panin 2000; Legesse et al. 2008). Some studies (Dick et al. 2008; Tologbonse et al. 2011) indicate that different adaptation methods to climate change are applied by sheep and goats farmers at different agro-ecological zones, but these studies failed to identify the determinants of each adaptation method used by each farmer located at each agro-ecological zone. This study, therefore, seeks to analyze the determinants of choices of adaptation strategies to climate change by sheep and goat farmers in the Southern and Central Tigray Zones, North Ethiopia.

## Methods

### Description of the study area

This study was carried out in three districts (Kolla-Tembein, Alaje and Ofla) located in the Tigray Regional State, Northern Ethiopia. Kolla-Temben is situated in Central Tigray zone; Alaje and Ofla are in Southern Tigray zone. The Kolla-Temben, Alaje and Ofla districts represent lowland, midland and highland agro-ecological settings, respectively. Geographically, the Southern Tigray zone is located at 12° 57′ 37.2″ (12.9603°) N latitude and 39° 31′ 41.9″ (39.5283°) E longitudes with average elevation of 2664 meters above sea level. Whereas the Central Tigray zone is located at 13° 47′ 6″ (13.78507°) N latitude and 38° 49′ 14″ (38.82054°) E longitude with average elevation of 1197 m above sea level.

### Sampling procedure

Purposive sampling method was employed to select three districts namely Kolla-Tembien, Alaje and Ofla; which represents low land, mid land and high land agro-ecological setting respectively. The districts selected have potential for small ruminant farming and sheep and goats have been inhabited in these districts since long ago.

A representative sample size was estimated at 95 % confidence level and below 1 % error commitment, as shown below (Chand et al. 2012):

$$n = \frac{{NZ^{2} P(1 - P)}}{{N \cdot e + Z^{2} P(1 - P)}}$$

where n = is the sample size, N = is the population size, Z = Confidence level at 95 %, Z = 1.96, P = Estimated population proportion (0.5), e = is the precision level (0.003).

Based on the sampling estimation made 318 sample households were selected out of the total 72,326 households. Again, out of 318 sample households; 118, 89 and 111 households were drawn from Kolla-tembein, Alaje and Ofla district, respectively. Sample size drawn from each district is proportional to targeted household population in each respective district as shown in Table 1.

**Table 1:** Sample size distribution by districts and agro-ecological zone

Districts	Agro-ecological zone	Target household population^a^	Sample size
Kolla-Tembien	Lowland	26,867	118
Alaje	Midland	20,081	89
Ofla	Highland	25,378	111
Total		72,326	318

^a^ *Source*: Central Statistics Agency of Ethiopia (CSA, 2007)

Households those having either sheep and/or goat herd obtained from the administrative office of each district was used as a sampling frame. The final sample households were selected from the sampling frame using systematic random sampling technique.

### Method of data collection

Both qualitative and quantitative data were collected for this study. Qualitative data were obtained using in-depth interviews that included group discussants and key informants, drawn from livestock experts, extension workers, district officials, and local leaders. Using household survey, primary data were obtained from the sampled respondents using semi-structured questionnaire (Additional file 1). The semi-structured questionnaire (close-ended multiple choice and open-ended type questions) was used to generate quantitative data on household characteristics, socio-economic parameters, marketing, institutional, and educational features of the sheep and goats farmers through interview, and sample household heads were the unit of analysis.

### Data analysis

Data were analyzed using STATA software version 11. Descriptive statistical tools like mean, percentage, minimum and maximum were employed to analyze, describe and summarize respondents’ socioeconomic, cultural, environmental and climate related variables.

### Econometric analysis

Multivariate probit model was employed to investigate the factors that determine the choice of adaptation strategies. OLS (Ordinary Least Square) model was also applied to demonstrate the effect of each adaptation strategy on income generated from the sales of sheep and goats.

Farmers’ adaptation activities to respond to climate change can be influenced by various factors, including household income, market, culture, and institutions. This study analyzed various factors that influence the producers of sheep and goats in choosing context-based adaptation methods to cope climate change effects. Farmers rearing sheep and goats can carry out many adaptation actions as long as their activity provides them a certain level of benefits. The adaptation choice that each farmer has to make can also be based on the resources they possess.

Identification of each factor that influences the behavior of farmers is very important. Although the multinomial probit can be used to measure the set of adaptation choices being applied by sheep and goats producers, its limitation is difficult to make interpretations for the simultaneous influences of explanatory variables on each outcome variable (endogeneity problem cannot be addressed using multinomial probit). This is because the local adaptive choices practiced by the farmers are either substitutive or supplementary of one another. Even if the univariate probit model is possible to estimate the adaptive choices of farmers on the available alternative measures, it is prone to bias due to neglecting the common factors that are not observable and unmeasured. In this case, a separate measurement using probit model never shows the relationships among various adaptation choices.

The multivariate probit model is appropriate to handle such measurement problems. It also allows the user to produce more than one equation with correlated disturbances, thereby enabling examination of the relationships among the outcome variables. During estimation, the adaptation choices of dependent variables in the multivariate model do not have negative values and hence, the error terms could be correlated to several predictors. Unlike in the ordinary least square method (OLS), the assumption of mean zero is senseless in the use of a multivariate model. By addressing the correlations of the error terms among unobserved adaptation choices, the multivariate model ensures statistical efficiency in the estimations of available choices as shown below (Lin et al. 2005).

$$\begin{aligned} {{Y}}_{i} &= 1\quad if\;X^{'} \beta_{i} + \varepsilon_{i} > 0 \hfill \\ Y_{i} &= 0\quad if\,X^{'} \beta_{i} + \varepsilon_{i} \le 0,\quad i = 1,\;2,\;3, \ldots ,n \hfill \\ \end{aligned}$$

where *Y*_*i*_ is a vector of dependent variables (each serves as adaptation choice), *X*ʹ is a vector of explanatory variables, *β*_*i*_ is a vector of coefficients, *ε*_*i*_ is a random error term and *n* is number of observations with zero means and unitary variance.

Exploring determinants of adaptation to cope with climate change risk alone will not provide full information. Thus, it is critical to investigate advantage of the strategies farmers consider fitting to adapt climate change. Accordingly, the study tried to show the effect of adaptation practices, currently used by sheep and goats farmers, on farmers’ livelihoods. Hence, income from the sale of sheep and goats was used as a dependent variable.

In the first instance, Heckman model was regressed to examine the effect of each adaptation strategy on income from the sale of sheep and goat production. Due to the unobservable nature of the dependent variable for some observations, the outcome variable was not observed for all respondents, but selection bias was not the problem. Because an inverse Miller ratio was not significant in a Heckman two-stage estimation method, implying that applying the OLS model is appropriate.

Income from the sale of sheep and goats is given by the equation as:

$${{Y}}_{i} = X^{'} \beta_{i} + \varepsilon_{i} > 0$$

where Y_i_ is the individual household’s income obtained from sales of sheep and goats, Xi is a vector of observable factors that affect the level of income from sheep and goats market and $\varepsilon_{i}$ is the error term.

### Dependent and independent variables

#### Dependent variables

The dependent variables included in the analysis are the adaptation strategies adopted by sheep and goat farmers and income from the sale of sheep and goats. The most common adaptation strategies identified during focus group discussion and key informant interviews were feeding the sheep and goats at home (home feeding), provision of shade during cold and warm season, having crossbred animals and marketing during shocks.

#### Independent variables

Independent variables include in the analysis are socio-economic, institutional, and environmental factors. Specifically, desired variables were sex and age of the household head, household income, marital status, access to credit, educational status of the head, family size, farm size, agro-ecological zones, herd size, access to credit, access to extension service, access to information on climate change, farming experience, number of household in one village, and distance to main market. Independent variables are clearly mentioned in Table 2.

**Table 2:** Description of variables included in the analysis

Independent variables	Variable type	Variable measurement	Mean	SD
Sex of the head	Dummy	1 if male, 0 otherwise	0.773	0.421
Age of the head	Continuous	Year	43.405	9.855
Marital status	Dummy	1 if married, 0 otherwise	0.789	0.408
Family size	Continuous	Number	5.405	1.782
Land size	Continuous	Hectare	0.529	0.425
Herd size	Continuous	Total Livestock Unit (TLU)	5.532	4.200
Access to info.	Dummy	1 if there is access, 0 otherwise	0.984	0.124
Year of production	Continuous	Number	11.639	8.203
Number of households in one village	Continuous	Number	487.9	220.14
Extension assistance	Dummy	1 if household gets ext.assi. 0 otherwise	0.99	0.096
Credit access	Dummy	1 if there is access, 0 otherwise	0.927	0.259
Distance to mkt	Continuous	Km	4.1173	5.902
High land	Dummy	1 if respondent from highland, 0 otherwise	0.349	0.477
Low land	Dummy	1 if respondent from lowland, 0 otherwise	0.371	0.483
Mid land(base category)	Dummy	1 if respondent from midland, 0 otherwise	0.279	0.449
Monthly consumption	Continuous	Birr (1 USD = 19.73 Birr)	1124.1	929.64
Edu1(base category)	Dummy	1 if illiterate, 0 otherwise	0.345	0.476
Edu2	Dummy	1 if informally literate (read and write), 0 otherwise	0.154	0.361
Edu3	Dummy	1 if primary school completed, 0 otherwise	0.443	0.497
Edu4	Dummy	1 if secondary school completed, 0 otherwise	0.047	0.212
Edu5	Dummy	1 if above secondary, 0 otherwise	0.009	0.096

## Results and discussion

### Socio-economic and institutional characteristics of the households

Socio-economic and institutional characteristics of the households are shown in Table 3. Three hundred eighteen households in three agro-ecological settings were enrolled in this study. Two hundred forty five (77.0 %) were male-headed households; whereas the remaining 73 (23.0 %) were female-headed. Household heads had a mean age of 43 years, and this ranged between 21 and 72 years. Education has an important effect on the choice of adaptation strategies. This is because educated individuals are expected to be exposed to better information about climate change; thereby he/she chooses compatible adaptation strategies. As shown in Table 3, 126 (34.6 %) were illiterate (at least they cannot read and write) and 18 (5.6 %) respondents completed secondary school and above. A significant number of the households (92.8 %) had access to credit, and over 60 % participated in off-farm activities. Particularly, the number of off farm participants in the midland agro ecological zone (Alaje district) was found to have the smallest share as compare to the two agro-ecological zones (Table 3). As confirmed by key informants and group discussants, most farmers in Alaje district have fertile farmlands and better opportunity to grow crops and vegetables using irrigation practices. This allowed farmers to stay in own farming instead of pursuing off farming. Income at household level is also an important variable, but under reporting of income by respondents is also expected. Because people are less willing to reveal their income compared to their expenditure. Thus, to control this variable expenditure was used as a proxy for income in this study.

**Table 3:** Socio-economic and institutional characteristics of sheep and goat farmers

Variables	Agro-ecological setting			Total
	Highland	Midland	Lowland	
*Sex head*				
Male (1)	54 (48.6)	82 (92.1)	109 (92.4)	245 (77.0)
Female (0)	57 (51.4)	7 (7.9)	9 (7.6)	73 (23.0)
*Education*				
Illiterate (1)	53 (47.7)	21 (23.6)	36 (30.5)	126 (39.6)
Informally literate (2)	29 (26.1)	16 (17.9)	4 (3.4)	49 (15.4)
Primary school (3)	22 (19.8)	48 (53.9)	71 (60.1)	141 (44.3)
Secondary school (4)	5 (4.6)	4 (4.5)	6 (5.0)	15 (4.7)
Above Secondary school (5)	2 (1.8)	0 (0)	1 (0.84)	3 (0.9)
*Access to credit*				
Yes (1)	98 (88.2)	86 (96.6)	111 (94.1)	295 (92.8)
No (0)	13 (11.7)	3 (3.4)	7 (5.9)	23 (7.2)
*Off-farm participation*				
Yes (1)	74 (66.7)	18 (20.2)	97 (82.2)	189 (59.4)
No (0)	37 (33.3)	71 (79.8)	21 (17.8)	129 (40.6)

### Farmers’ perceptions on climate change

**Fig. 1:**
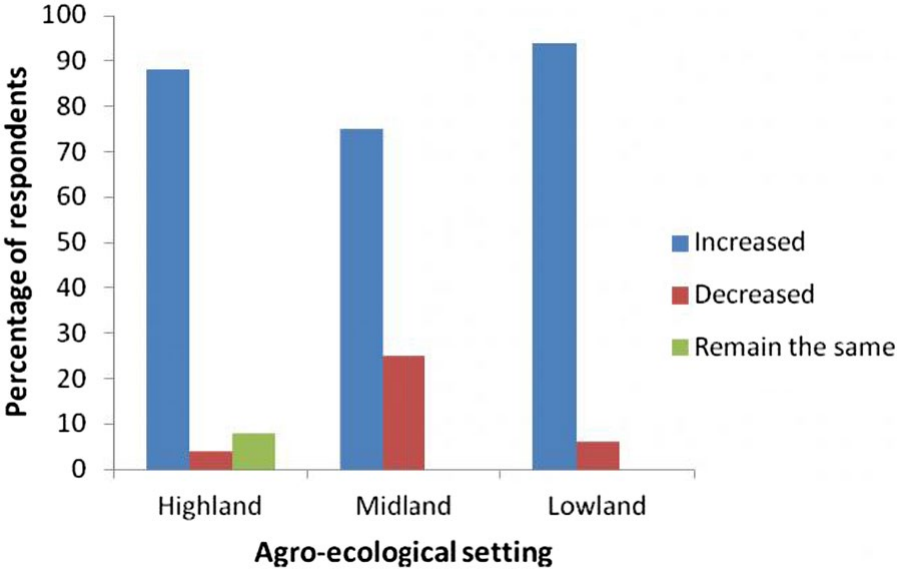
Farmers’ response about climate change through change in temperature. It indicates the change in temperature due to climate change as reported by farmers’ from three different agro-ecological settings of Southern and Central Tigray Zones

**Fig. 2:**
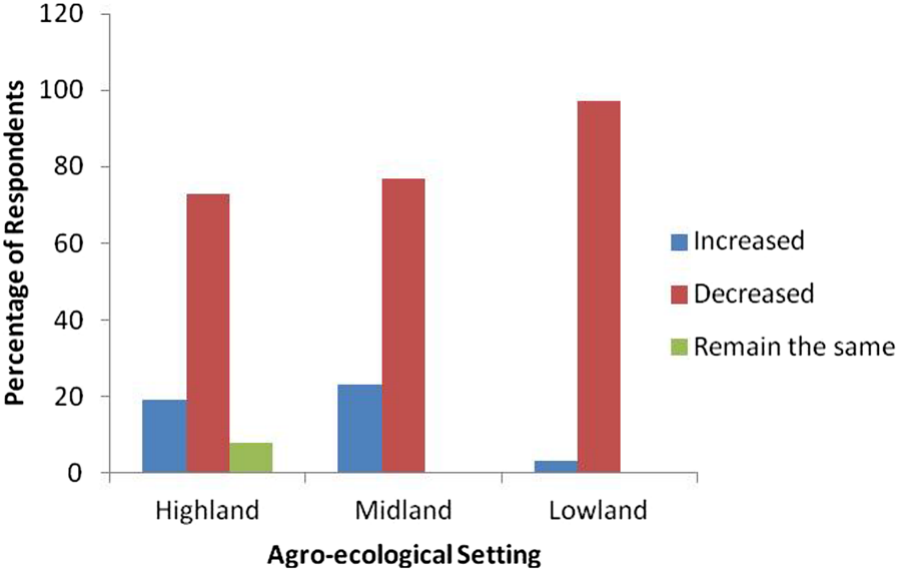
Farmers’ response about climate change through change in rainfall amount. It indicates the change in rainfall amount due to climate change as reported by farmers’ from three different agro-ecological settings of Southern and Central Tigray Zones

Farmers were asked about their perception whether climate is changing or not over the last 10 years. Most of the respondents (96.0 %) perceived that climate change is indeed occurring. Among climate change indicators, temperature and rainfall were considered as parameters for the analysis. The responses from respondents in relation to changes in temperature and rainfall across three agro-ecological zones are depicted in Figs. 1 and 2, respectively. Most of the respondents acknowledged that there is rise in temperature and decline in rainfall amount.

Eighty-eight percent and 73 % of the respondents from the high-land agro-ecological zone observed that the temperature was rising and the rainfall level was declining in the last 10 years, respectively. A few respondents (8 %) in this agro-ecological zone had reported that there is no change both in temperature and rainfall amount. Similar to the high-land agro-ecological zone, respondents in mid land consisted large proportion in reporting rise in temperature (75 %) and decline in rainfall amount (77 %). In the same line, in lowland agro-ecological zone, respondents perceived that the temperature was increasing (94 %) whereas the rainfall amount was declining (97 %) over the last 10 years.

Farmers’ response towards perception on climate change is consistent with other studies. Studies conducted in Ethiopia by Deressa et al. (2008) and Mengestu (2011) reported that the temperature is rising and rainfall amount is decreasing due to climate change. Studies conducted in other African countries like South Africa (Mandleni and Anim 2011a), Ghana (Kemausuor et al. 2011), and Nigeria (Apata, 2011) also documented similar findings with this study on farmers’ perception about climate change.

### Adaptation strategies to climate change pursued by farmers

The distribution of adaptation strategies used by sheep and goat farmers in response to climate change is shown in Table 4. The most common adaptation strategy is marketing during shock. A total of 307 (96.5 %) farmers were found to use marketing during shock as a climate change adaptation strategy. This practice enabled farmers to sell their sheep and goats during extreme weather events because animals were unable to resist long dry periods due to deficiency of feed and water. However, this has its own drawback, as animals will not be fetching good prices; ideally it is recommended that farmers participate in the normal time market. The second most commonly used adaptation strategy by the farmers is home feeding. Out of the total respondents, 285 (89.6 %) of them practiced this adaptation strategy. As reported by key informants and group discussants, this was mainly because of the introduction of area enclosures in almost all communal lands of villages by which farmers were obliged to feed their animals at home. As shown in Table 4, provision of shade during extreme weather events, hot and cold season, was the least practiced adaptation option to cope with climate change effects. This may be because of incidental expenses related to building houses and preparation of bedding that require to incur the high cost of materials and skilled human capital.

**Table 4:** Distribution of adaptation options used by sheep and goats farmers

Adaptation options	Agro-ecological settings			Total
	Low-land	Mid-land	High-land	
Provision of shade	27 (22.8)	48 (54.0)	57 (52.7)	132 (41.5)
Home feeding	103 (87.2)	86 (96.6)	96 (88.8)	285 (89.6)
Use of crossbred animals	63 (53.3)	43 (48.3)	67 (62.0)	173 (54.4)
Marketing during shock	117 (99.0)	85 (95.5)	105 (97.0)	307 (96.5)

Numbers in parenthesis indicate percentage

The distribution of adaptation strategies by agro-ecological settings is also presented in Table 4. In all the three agro-ecological settings, marketing during climate shock is the most commonly used option. On the other side, providing shade during hot and cold season is the least practiced adaptation practice in all the study sites. The table clearly shows that farmers exercising provision of shade in lowland agro-ecological zones consisted of small proportion (22.8 %) as compared to those of in the mid-land (54 %) and highland (52.7 %) regions. Since goats are relatively tolerant of high temperature and are better able to survive in the lowland, farmers in this area may be reluctant to engage in putting up shade, compared to those in the midland and highland areas who mainly rearing sheep.

### Determinants of choice of adaptation practices by sheep and goat farmers

Prior to the main estimation, pre-estimation, tests were undertaken. Multicollinearity was tested using Variance Inflation Factor (VIF) and Contingent Coefficient (CC) for continuous and discrete explanatory variables, respectively (Additional file 2). VIF for all continuous variables were <10, and CC for all discrete variables was <0.75, which indicate multicollinearity is not a serious problem in the model estimation (Gujarati 2004; Rabe-Hesketh and Everitt 2004). The result of multivariate probit model is presented in Table 5. Although education was presumed to have an important effect on the choice of adaptation strategies while education was insignificant in adopting adaptation strategies (Table 5). This could be the reason that educated individuals in the study area are engaged in searching off farm job. Therefore, they are less likely to participate in farming practice in which adaptation is required. Only those variables whose coefficients are statistically significant at 1 and 5 % probability levels were discussed.

**Table 5:** Results of multivariate probit model for determinants of adaptation choices

Independent variables	Dependent variables							
	Home feeding		Crossbred		Marketing		Shade	
	Coeff.	P value	Coeff.	P value	Coeff.	P value	Coeff.	P value
Sex	−0.8021*	0.097	−0.2438	0.450	−0.1195	0.848	−0.0901	0.772
Age	0.0160	0.280	−0.0142	0.159	0.0318	0.325	−0.0209*	0.053
Marital status	0.8064*	0.068	0.5333*	0.076	0.2756	0.640	0.2647	0.368
Family size	0.0020	0.978	0.0509	0.356	−0.0554	0.681	0.1084*	0.052
Land size	0.4527	0.283	0.0140	0.944	0.1522	0.849	−0.1722	0.492
Herd size	0.0008	0.979	0.0439	0.100*	0.0459	0.514	0.0067	0.780
Access to info.	1.7645**	0.013	1.7009**	0.027	3.1643***	0.000	0.9316	0.184
Year of production	−0.0267	0.158	0.0310**	0.014	0.0048	0.887	0.0323***	0.009
No. households in one village	0.0006	0.340	−0.0015***	0.001	0.0007	0.428	−0.0010**	0.031
Credit access	−0.4864	0.418	−0.4548	0.211	−3.6721	0.988	−0.0986	0.792
Distance to mkt	−0.0398***	0.007	0.0020	0.879	0.0180	0.739	0.0116	0.373
Highland	−1.1812**	0.017	1.7441***	0.000	0.6275	0.380	0.6206**	0.043
Lowland	−1.4700***	0.000	0.0101	0.962	0.6501	0.270	−0.7218***	0.001
Monthly consumption	−0.4818**	0.023	−0.4502***	0.001	−0.2690	0.381	0.0518	0.712
Edu2	0.2731	0.455	−0.0194	0.940	0.1190	0.823	0.0128	0.959
Edu3	0.0127	0.966	−0.0184	0.932	0.0724	0.881	0.0262	0.901
Edu4	−0.3680	0.470	−0.3914	0.355	0.7316	0.562	−0.0210	0.962
Edu5	4.2013	0.993	5.0015	0.989	3.1296	0.996	5.2491	0.987

*, ** and *** are at 10, 5 and 1 % level significant respectively

#### Access to information

This variable represents sources of information required to make the decision to adapt to climate change such as TV, radio, magazine, newspaper, personal observation, development agents, etc. An individual exposed to climate information is more likely to take an immediate action to cope with risks related to climate change. The model result shows that access to information has positive and significant impact on home feeding, use of crossbred animals, and marketing during shock (Table 5). Many studies also reported strong positive relationship between access to information and adaptation (Deressa et al. 2008; Asayehegn 2012; Di Faclo et al. 2011; Tazeze et al. 2012; Balew et al. 2014).

#### Farming experience

Farming experience in the rearing of sheep and goats was one of the explanatory variables thought to affect adaptation strategies to climate change. Farming experience positively and significantly affects the choice of having crossbred animals and shading adaptation practices. This effect suggests that farmers with longer periods of farming experience were more likely to understand climate change and its negative consequences and are more willing to respond to climate change effects through implementing different adaptation practices. In addition, farmers with experience observe changes over time and compare such changes with the current climatic conditions, which enable them to respond to climate change. This result is consistent with other numerous studies (Dhakal et al. 2013; Mabe et al. 2014; Obayelu et al. 2014).

#### Number of households in one village

The coefficient of this variable has a significant and negative relationship with the likelihood of choosing adaptation measures; crossbred and provision of shade. In the case of shading as adaptation practice, increase in number households in one village may result in shortage of land. Thus, farmers cannot have enough places to prepare shade for their animals.

#### Distance to market (km)

The model result shows that as the distance to market increases, the probability of choosing the adaptation practice to feed the animals at home decreases. The analysis shows statistical significance at the 5 % probability level. Households far from the main market may not get supplementary feed easily and prefer to let the animals graze. Market was one means of exchanging information with other farmers, and it provides an opportunity for sharing experiences on adaptation to climate change. Similar findings were also reported by (Hassan and Nhemachena 2008; Tazeze et al. 2012; Balew et al. 2014).

#### Highland agro-ecological zone

As expected, different farmers live in different agro-ecological settings, take up different adaptation options (Deressa et al. 2008; Tazeze et al. 2012). This explanatory variable was found to have a significant effect on the provision of shade, having crossbred animals, and home feeding. The model showed a positive relationship of adoption to having crossbred animals and shading adaptation practices, but not for the home feeding practice. This implies that being a resident in highland agro-ecological zone, as compared to that of midland, increases the probability of having crossbred animals and implementing shading practice; whereas it reduces the probability of using home feeding adaptation practice.

#### Lowland agro-ecological zone

Farmers living in lowland agro-ecological zone are less likely to practice shading management and to feed their sheep and goats at home. This explanatory variable affects the probability of choosing home feeding and provision of shade as an adaptation strategy at 1 % significance level. This could be the reason that goats are resistant to dry season are dominant in lowland agro-ecological zone.

#### Monthly consumption (income)

The study found that household income has a negative and significant impact on the choice of adaptation options having crossbred animals and home feeding. This may be because higher income farmers may be less risk averse, and as a result, they may not pay for adaptation measures against climate change. A study by Mandleni and Anim (2011b) has shown that non-farm income decreased the likelihood of adaptation measures. On the other hand, contradicting findings were also reported in studies by Deressa et al. (2008), Sahua and Mishrab (2013), Getachew et al. (2014), Mabe et al. (2014), where household income is positively associated with adaptation measures.

### Do adaptation strategies have contribution on income from sheep and goat sales?

The result (Table 6) shows that annual income from the sale of sheep and goats was positively related to farmer’s adaptation practices. Home feeding and having crossbred animals affect the income from the sale of sheep and goats at 1 % significance level. Practicing home feeding and having crossbred animals increased the revenue from the trade of sheep and goat by 1877 and 1182 birr^1^ respectively. Other variables such as herd size and access to credit have positive and significant effects on the income. As the herd size increased, animals offered to the market also increased, which results in additional revenues. As the herd size increased by one unit, the sale of sheep and goats increased by 498 birr. Access to credit also has a positive influence on the sales of sheep and goats. This may be because access to credit reduces the financial burden to purchase animal feed and other farm inputs, which boost their agricultural production overall.

**Table 6:** Results of OLS model for determinants of income from the sales of sheep and goat

Independent variables	Coefficient	P value
Home_feeding	1877.711***	0.007
Cross bred_animal	1182.326***	0.006
Shade	1182.326	0.142
Sex	688.3208	0.289
Age	22.0632	0.303
Farm size	−166.2574	0.169
Land_size	91.93275	0.823
Herd size	498.7345***	0.000
Farm association	−1982.854***	0.001
Extension assistance	1556.691	0.404
Credit acess	1841.97**	0.040
mkt_km	4.680807	0.870
High_land	179.9796	0.801
Low_land	−1224.172**	0.044
Monthly consumption	352.5096	0.320
edu2	−482.141	0.424
edu3	−480.5437	0.314
edu4	−629.2862	0.518
edu5	−2291.116	0.412
_cons	−3748.746	0.233

** and *** are at 5 and 1 % significance level respectively

Farming in the lowland agro-ecological setting and involvement in farm associations affects the outcome variable negatively, though the latter was expected to affect the outcome variable positively. This is because, as key informants interview reveal, farm associations build social-capital that supports farmers in providing different technical guidance and advice about agricultural production and overall rural development. Long-dry season is one of the features of lowland agro-ecological zone as compared to other agro-ecological zones, which affects animal feed to be scarce and decreases its nutritive value. Hence, farmers in lowland agro-ecological settings are less competitive in the market of sheep and goat, which indicates that revenue from the sales of sheep and goats, is quite low. Assuming other factors constant, living as a farmer in lowland agro-ecology and involved in farm association decreases the sale of sheep and goats by 1224 and 1982 birr, respectively.

## Conclusion and recommendations

Findings from *Ofla*, *Alaje* and *Kola*-*Tembien* suggest that more than 96 % of local farmers were able to perceive the adverse effects of climate change. They apparently noticed that climate change drastically reduced the amount of rainfall, which evidently exhibited in terms of occurrence of frequent drought with its immediate consequences on loss of their livestock and crop productivity. In the due course of responding the negative effects of climate change, producers of small ruminants continued to pursue multiple adaptation methods. Field -based assessments on indicators of multiple adaptation choices were conducted and the estimated results indicated that nearly 96 % of the farmers were found to use marketing. During drought periods, farmers used to sell their livestock because of fear of lack of natural grazing and animal feed.

The findings from multivariate probit model revealed that the farmers’ choice of adaptation strategies were statistically and significantly affected by factors such as access to information, farming experience, distance to main market, household income, agro-ecological zone and number of households in a village. Moreover, results from OLS model revealed that home feeding strategy (the strategy of keeping and feeding animals at home) was recently getting adopted by farmers. As reasoned out by key informants, farmers chose to pursue zero-grazing because they have already experienced that the use of communal water sources and free grazing were the sources of communicable diseases. It was also found that the strategy to access to cross bred animals was an important factor, which positively and significantly associated to the household income level.

However, the emphasis of this study was mainly to identify the possible adaptation choices applied by small ruminant producers. Environmental effects of producing small ruminant animals are beyond the scope of this study. Thus, we suggest further investigation on issues of rangeland capacity to accommodate herds of sheep and goats sustainability. Considering the above findings and shortfalls, it is suggested to design early warning policy systems that aim to make the locals aware of future climate variability and potential shocks so that they can take proactive steps to use varying approaches that best fit to different agro-climatic conditions.

## Additional files

10.1186/s40064-016-3042-3 Questionnaire.
10.1186/s40064-016-3042-3 Variance Inflation Factor (VIF) and Contingent Coefficient (CC) for continuous and discrete explanatory variables.

## Footnotes

1 1USD was equivalent to 19.73 Ethiopian Birr when the data for this study was collected in July 2014.
