# Supplementary material for: Determinants of adaptation choices to climate change by sheep and goat farmers in Northern Ethiopia: the case of Southern and Central Tigray, Ethiopia
Source: Springerplus. 2016 Oct 1;5(1):1692. doi: 10.1186/s40064-016-3042-3 (PMC5045456; doi:10.1186/s40064-016-3042-3)
Supplement: Supplementary file 1 — 10.1186/s40064-016-3042-3 Questionnaire. [file 40064_2016_3042_MOESM1_ESM.docx]

**Household Survey Questionnaire**

**Dear Respondents:**

This questionnaire is prepared to collect data for analyzing determinants of adaptation choice to climate change by small ruminant producer in southern and central Tigray. The information given to us is highly privileged and every information’s which will be provided by you is kept confidential.

Thank you in advance for your cooperation!

#

Questionnaire number …………………………...............................................................……….

Name of the Interviewer…………………………...............................................................……

Woreda/District………………….….................................................................................................

Tabia/Village……………..........................................................……......…………………………..

Date of interview……….................................................................................................…………...

**I- Demographic information about the Household (HH)**

1- Name of Household head……......................…………...……....………………………………

2- Gender of the Household head: 1. = Male 0.= Female

3- Age of the Household head: ........................................................................................................

4- Household head marital status:

1. Single 2. Married

3. Divorced/Separated 4. Widow

5- Religion of the household

1.Orthodox 4.Muslim

2.Catholic 5.Protestant

3. Others

6- Educational level of household head:

1.Illiterate

2.Tertiary

3. Primary

4.Secondary

5.Above secondary

7- Number of family Members:

1. Males 2. Females Total

8- Major occupation of the household head:

1. Farmer 2. Daily laborer 3. Business 4. Employed 5. Self employed - mason/carpenter/ artisan

6. Other (list down if any)………………………………………………....………………………

........................................................................................................................................................................................................................................................................................................................

**II- Assets, activities and income of the household**

9- Do you own land? 1= Yes 0 = No

10- If “Yes”, what is the total size of your land?..................................................... in Tsimad (ha)

11-How did you get the land:

1= Purchase 2= Rent 3= Inheritance

5= Others (list down if any)………........………………………………………..............................

............................................................................................................................................................

............................................................................................................................................................

12- What are the major crops grown on your farm ? (list them down)

………………………......................................................................................................................................................………………………………………………………........…………………...

..........................................................................................................................................................

13. How much (in Quintile/ha) do you harvest per season and/ or year?

...........................................................................................................................................................

14- Is your annual production sufficient for household consumption throughout the year?

1= Yes 0 = No

15- If “No”, how do you manage the deficit? ...................................................................................

…………………………………………………………………………………………………....................................................................................................................................................................

16- Do you own livestock (practice animal husbandry)?

1= Yes 0 = No

17- If “Yes”, what types and number of livestock do you have? (if “No”, skip to Q17)

| No | Livestock type | Number | Value in Ethiopian birr/individual | Total value in Ethiopian Birr | Remarks |
| --- | --- | --- | --- | --- | --- |
| 1 | Sheep |  |  |  |  |
| 2 | Goat |  |  |  |  |
| 3 | Poultry |  |  |  |  |
| 4 | Cow |  |  |  |  |
| 5 | Ox |  |  |  |  |
| 6 | Donkey |  |  |  |  |
| 7 | Camel |  |  |  |  |
| 8 | Others |  |  |  |  |

18. For what purpose you employed your sheep and goat herd?

1. For household consumption only

2. For earning income from sell of sheep and goat

3. For both (1 and 2)

19- Are you involved in some off-farm (extra) activities? 1= Yes 0 = No

20. If "Yes", to the above question, list them down:

………………………………………………....………………………….......................................

............................................................................................................................................................

...........................................................................................................................................................

....................................................................................................................................................................................................................................................................................................................

21- How much do you obtain from sale of your one sheep and goat herds?

1. Sheep....................................... Ethiopian birr

2. Goat .........................................Ethiopian birr

22. How much do you earn from off-farm activities?

...................................................................................................................................Ethiopian birr.

23. How much do you spend for household consumption per month?

…………………………………………………………………………………..… Ethiopian birr.

24. Annual Household income from different activities:

24.1) Trade in Livestock ____________

A) Cattle ____________

B) Sheep ______________

C) Goat ________________

D) Poultry _______________

E) Milk _________________

F) Others (specify) ________________

24.2) Wage ________________

24.3) Trade in grain ___________________

24.4) Trade in Handicraft/weaving/hair dressing ___________________

24.5) Selling charcoal and wood ________________________

24.6) Others (specify) _____________________________

.

**C- Attitude/ Farmers' perception about climate change**

25- Do you think the climate is changing?

1.= Yes 0. = No

26- If “Yes”, to Q#21, what do u think causes climate change?

| Cause of climate change | 5=highly Agree | 4=Agree | 3=Average | 2=Disagree | 1=Highly Disagree |
| --- | --- | --- | --- | --- | --- |
| a) Natural Variability |  |  |  |  |  |
| b) Deforestation |  |  |  |  |  |
| c) Overgrazing |  |  |  |  |  |
| d) Population growth |  |  |  |  |  |
| e) Urbanization |  |  |  |  |  |
| f) Industrialization |  |  |  |  |  |
| g) Wetland degradation |  |  |  |  |  |
| h) Transportation (more vehicles) |  |  |  |  |  |
| i) Agricultural expansion |  |  |  |  |  |
| j) Energy Production |  |  |  |  |  |
| k) Mining activities |  |  |  |  |  |
| l) Poor solid waste management |  |  |  |  |  |
| m) Others.................................... |  |  |  |  |  |

27. What are the indicators (impacts) of a changing climate?

| Evidence of climate change | 5=Highly Agree | 4=Agree | 3=Average | 2=Disagree | 1=Highly Disagree |
| --- | --- | --- | --- | --- | --- |
| 1.a) Rise in Temperature |  |  |  |  |  |
| b) decrease in Temperature |  |  |  |  |  |
| c) No change in Temperature |  |  |  |  |  |
| 2.a) Increase in Precipitation |  |  |  |  |  |
| b) Decline in precipitation |  |  |  |  |  |
| c) No change in precipitation at all |  |  |  |  |  |
| 3.Changes in seasonal patterns |  |  |  |  |  |
| 4.Changes in the timing of rainfall |  |  |  |  |  |
| 5.Reduced crop yields (crop failure) |  |  |  |  |  |
| 6.Limited availability of water |  |  |  |  |  |
| 7.Increased incidence of pest/disease attack |  |  |  |  |  |
| 8.Frequent occurrence of droughts |  |  |  |  |  |
| 9.Reduction in soil fertility |  |  |  |  |  |
| 10.Occurrence of floods |  |  |  |  |  |
| 11.Reduced indigenous biodiversity |  |  |  |  |  |
| 12.Death of livestock |  |  |  |  |  |
| 13. Increased hunger and famine |  |  |  |  |  |
| 14. Loss of cultivable land |  |  |  |  |  |
| 15. Increased food prices |  |  |  |  |  |
| 16. Loss of farm household income |  |  |  |  |  |
| 17. Conflict over scarce resources (water...) |  |  |  |  |  |
| Others ................................................ |  |  |  |  |  |

**D- Adaptation practices to climate change**

28- Do you practice any climate change adaptation option on your small ruminant herd?

1= Yes 0= No

29- If "Yes", to Q#28, answer the following questions.

30. What are the different adaptation practices employed on your small ruminant herd?

| Adaptation practices | 1=YES | 0=NO | Year of starting the practice |
| --- | --- | --- | --- |
| 1.Provision of housing |  |  |  |
| 2.Cleaning of the house |  |  |  |
| 3.Good health care by inviting vet. doctor |  |  |  |
| 4.Provision of shade for animals during day |  |  |  |
| 5.Traditional health care |  |  |  |
| 6.Good feed and water provision |  |  |  |
| 7.Provision of feed supplements |  |  |  |
| 8.Keeping and feeding the animal during rainfall and dry season |  |  |  |
| 9.Irrigation of pasture during dry season |  |  |  |
| 10.Use of hybrid animals (drought resistance) |  |  |  |
| 11.marketing during shock |  |  |  |
| 12. No Adaptation at all |  |  |  |
| 13.Others....................................................... |  |  |  |

31.What are the opportunities/benefits of applying the above-mentioned adaptation options?

………………………........................................................................................................................

........................................................................................................................................................................................................................................................................................................................

32. Do you have access to climate data and information? 1= Yes 0 = No

33. What is your source of information?

1. Television

2. Radio

3. Personal observation

4. Development agents

5. Farmer Association

6. Others ……………………………………………………………….

34. Do you get any assistance from extension office? 1= Yes 0 = No

35. Do you have access to improved technologies like;

- Use of fertilizers 1= Yes 0 = No
- Improved seed varieties 1= Yes 0 = No
- Pesticides 1= Yes 0 = No
- Mechanization (tractor, plough) 1= Yes 0 = No

36. Do you get any loan (credit) for your farm activities? 1= Yes 0 = No

37. How far ( distance in Km) do you have to travel to the Main Market?

.......................................................................................................................................................

**E- Barriers /Constraints faced by small farmers in the adoption of various adaptation practices**

38.What challenges (constraints) do you face in using the various adaptation practices?

1. …………………………………………………………........................……………………
2. ………………………………………………………….......….................…………………
3. ……………………………………………………………....................……………………
4. ................................................................................................................................................
5. ................................................................................................................................................
6. ................................................................................................................................................

39. Do you think climate change can be tackled? 1= Yes 0 = No

40. If "Yes", what do you think needs to be done to address climate change?

........................................................................................................................................................................................................................................................................................................................

............................................................................................................................................................

**Interview Schedule for Group Discussants**

1. What type of adaptation strategies are commonly used in your locality by small ruminant farmers?

___________________________________________________________________________________________________________________________________________________________________________________________________________________________________

1. Are there any opportunities that help farmers to cope climate change in your area?

_____________________________________________________________________________________________________________________________________________________________________________________________________________________________________________

1. Is there any support (financial, technical etc) from concerned bodies for farmers in order to help their effort during the use of adaptation mechanisms?

______________________________________________________________________________________________________________________________________________________________________________________________________________________________________

1. What will be the contribution of farm associations in using appropriate adaptation mechanisms?

_____________________________________________________________________________________________________________________________________________________________________________________________________________________________________________

**Thank you!!!**
