# Supplementary material for: Determinants of adaptation choices to climate change by sheep and goat farmers in Northern Ethiopia: the case of Southern and Central Tigray, Ethiopia
Source: Springerplus. 2016 Oct 1;5(1):1692. doi: 10.1186/s40064-016-3042-3 (PMC5045456; doi:10.1186/s40064-016-3042-3)
Supplement: Supplementary file 2 — 10.1186/s40064-016-3042-3 Variance Inflation Factor (VIF) and Contingent Coefficient (CC) for continuous and discrete explanatory variables. [file 40064_2016_3042_MOESM2_ESM.docx]

**Annex**

Table A 1: Variance inflation factor values of the continuous variables

| Variables | VIF | 1/VIF |
| --- | --- | --- |
| Farming experience | 1.61 | 0.619563 |
| Herd size | 1.52 | 0.657474 |
| Age | 1.48 | 0.675695 |
| Family size | 1.29 | 0.774800 |
| No of households in one village | 1.22 | 0.816910 |
| Land size | 1.17 | 0.856573 |
| Monthly consumption | 1.14 | 0.873482 |
| Distance to market | 1.07 | 0.934616 |
| Mean VIF | 1.31 |  |

Table A 2: Contingent coefficient values of the discrete variables

| variables | sex | marital status | Access info. | credit access | highland | lowland | edu2 | edu3 | edu4 | edu5 |
| --- | --- | --- | --- | --- | --- | --- | --- | --- | --- | --- |
| sex | 1.0000 |  |  |  |  |  |  |  |  |  |
| Martial status | 0.7265 | 1.0000 |  |  |  |  |  |  |  |  |
| Access.info | -0.0089 | -0.0033 | 1.0000 |  |  |  |  |  |  |  |
| Credit access | 0.1651 | 0.1237 | -0.0353 | 1.0000 |  |  |  |  |  |  |
| highland | -0.4944 | -0.3982 | -0.1196 | -0.1266 | 1.0000 |  |  |  |  |  |
| lowland | 0.2800 | 0.2532 | 0.0971 | 0.0386 | -0.5625 | 1.0000 |  |  |  |  |
| edu2 | -0.0984 | -0.0999 | -0.0161 | -0.0153 | 0.2174 | -0.2557 | 1.0000 |  |  |  |
| edu3 | 0.3517 | 0.3370 | 0.0619 | 0.0048 | -0.3614 | 0.2448 | -0.3809 | 1.0000 |  |  |
| edu4 | 0.0509 | 0.0058 | -0.0911 | -0.0524 | -0.0073 | 0.0133 | -0.0950 | -0.1986 | 1.0000 |  |
| edu5 | 0.0533 | 0.0504 | 0.0123 | -0.0983 | 0.0650 | -0.0076 | -0.0417 | -0.0871 | -0.0217 | 1.0000 |
